# Blistering barnacles: Space physiology in *The Adventures of Tintin*

**DOI:** 10.1113/EP092571

**Published:** 2025-02-26

**Authors:** Jacob P. Hartmann, Mathis B. Mottelson, Rasmus H. Dahl, Ronni R. Plovsing, Ronan M. G. Berg

**Affiliations:** ^1^ Centre for Physical Activity Research Copenhagen University Hospital – Rigshospitalet Copenhagen Denmark; ^2^ Department of Clinical Physiology and Nuclear Medicine Copenhagen University Hospital – Rigshospitalet Copenhagen Denmark; ^3^ Department of Biomedical Sciences, Faculty of Health and Medical Sciences University of Copenhagen Copenhagen Denmark; ^4^ Department of Internal Medicine Zealand University Hospital Roskilde Denmark; ^5^ Department of Radiology Copenhagen University Hospital – Rigshospitalet Copenhagen Denmark; ^6^ Department of Anaesthesia and Intensive Care Copenhagen University Hospital – Hvidovre Hospital Hvidovre Denmark; ^7^ Department of Clinical Medicine, Faculty of Health and Medical Sciences University of Copenhagen Copenhagen Denmark; ^8^ Neurovascular Research Laboratory, Faculty of Life Sciences and Education University of South Wales Pontypridd UK

‘That's one small step for a man, one giant leap for mankind!’, the American astronaut Neil Armstrong (1930–2012) famously stated when the Apollo 11 mission culminated in his first step on the Moon on 20 July 1969, marking a milestone in human space exploration. At the time, these efforts, rooted in international political tension, had influenced both science and popular culture for decades. This influence is also evident in *The Adventures of Tintin* by Georges ‘Hergé’ Remi (1907–1983), which centres on Tintin, the Belgian teenage reporter with his distinctive tuft of ginger hair, one of the most iconic and recognizable comic book characters of all time.

The 23 completed volumes of *The Adventures of Tintin*, published between 1929 and 1983, are among the most beloved European comics of the 20th century. Translated into >50 languages and having sold >200 million copies worldwide, the series remains a cultural phenomenon. Hergé’s distinctive *ligne claire* drawing style combines visual clarity with uncanny realism, in which the ageless Tintin, always accompanied by his faithful Wire Fox Terrier, Snowy, along with memorable characters such as Captain Archibald Haddock, Professor Cuthbert Calculus and the utterly useless detectives Thomson and Thompson, provide thrilling adventures set against the backdrop of the rapidly changing 20th century world. *The Adventures of Tintin* begin with *Tintin in the Land of the Soviets* (1929–1930) and, subsequently, take Tintin and his friends across every continent over the next 50 years, only to end in Tintin's own hometown of Brussels in the unfinished volume *Tintin and Alph‐Art* (1986), where his life seemingly meets an untimely end at the hands of his arch‐enemy, the ruthless movie mogul and criminal mastermind, Roberto Rastapopoulos. The stories explore a wide range of themes (arguably, in a rather Eurocentric and not always critical manner) regarding colonialism, racism, archaeology, occultism, totalitarianism, revolution, human trafficking, industrialization and technological development, to mention a few (Apostolidès, 2009; Horn, 1983; McCarthy, 2011). The French philosopher Michel Serres hailed *The Adventures of Tintin* as a masterpiece, declaring that ‘the work of no French novelist is comparable in importance or greatness’, while others have lauded it as ‘a powerful graphic record of the 20th century's tortured history’ and credited it with having ‘spearheaded the post‐World War II renaissance of European comic art’ (Horn, 1983). Indeed, *The Adventures of Tintin* has even been stated to conceal the very ‘secret of literature’ (McCarthy, 2011)!

The realism in *The Adventures of Tintin* owes much to the extensive research undertaken by Hergé and his team for each volume. Within the narratives of the series, the perceptions of the medical profession and scientific community of the time are not only vividly reflected but also comprise depictions of situations relevant to various fields of medicine and physiology. These include endocrinology, neuroscience, psychiatry and even infectious diseases (Castillo, 2011; Caumes et al., 2015, 2016; Cruz‐Culebras, 2021; Cyr et al., 2004; Förstl et al., 1990; Virk et al., 2024). Reading *The Adventures of Tintin* since our childhood in the 1980s and 1990s, when Tintin was an undisputed Pan‐European icon and a ubiquitous hero, we have noted further candid depictions of concepts directly relevant to human physiology. As we will explore, one of the most striking examples can be found in the Moon voyage duology, *Destination Moon* (1953) and *Explorers on the Moon* (1954), which is particularly pertinent to this space physiology special issue of *Experimental Physiology*.

In *Destination Moon*, Tintin and Captain Haddock barely have time to recover from a dramatic trip to the Middle East (*Land of Black Gold*, 1950) when they are summoned to Syldavia, a benevolent monarchy in Central Europe. There, their friend Professor Calculus (an extraordinary polymath with expertise spanning from botany, archaeology, biology and engineering to physics) has been headhunted to lead Syldavia's top‐secret space project. The mission's goal is to harness uranium‐based atomic energy to propel humanity into a new era of space exploration. However, the endeavour is fraught with danger, because aggressive foreign powers (most notably Borduria, a semi‐totalitarian neighbouring republic) plot to hijack this groundbreaking technology for sinister purposes. However, as literary critic Jean‐Marie Apostolidès highlights in *The Metamorphoses of Tintin*, this marks a departure from the classic ‘good versus evil’ dichotomy typical of Hergé’s earlier works. Instead, the narrative depicts a thematic struggle between ‘truth and error’ (Apostolidès, 2009), echoing challenges faced in science to this day (Drummond & Tipton, 2024).

One notable aspect of Hergé’s depiction of the Moon Rocket, particularly relevant to physiology, is its interior design, which places the crew in a prone position during take‐off. This concept draws inspiration from military aviation research conducted in the UK and Germany during the 1930s. Experiments with test pilots and centrifuge studies revealed that a reclined or prone position allowed individuals to tolerate higher gravitational (G)‐forces. Similar designs were proposed in early space exploration concepts but were later abandoned as spacecraft design evolved, owing to space constraints and operational challenges. In the story, the prone position should prevent gravitational loss of consciousness (G‐LOC), a condition resulting from blood pooling and the inability of the cardiovascular system to supply sufficient oxygenated blood to brain regions necessary for maintaining consciousness, and which usually sets in at accelerations of 7.0 G or higher in highly trained personnel (Whinnery & Forster, 2013). Indeed, and despite all precautions, Tintin and his fellow crew members pass out shortly after take‐off. By carefully scrutinizing text boxes and conversations and by inspecting available clocks in the ‘Moon voyage duology’, some uncanny facts emerge. In the following, we assume that the Moon Rocket travels with constant acceleration when powered by the auxiliary engine until reaching 805 km (500 international miles) of flight and when powered by the nuclear motor until reaching escape velocity of 11.3 km/s at 3219 km. Tintin regains consciousness 12 min after take‐off at 4023 km, and because the spacecraft travels ∼11.3 km/s between 3219 and 8047 km, it must have reached 3219 km after ∼10 min 48 s. Based on these time points, distances and velocities, the initial acceleration of the spacecraft is 13.5 m/s^2^. The auxiliary engine is turned off after 5 min 45 s while travelling at 4.7 km/s, and hereafter, the nuclear motor accelerates the spacecraft to 21.8 m/s^2^. Thus, assuming that the Moon Rocket flies straight up and accounting for the Earth's gravity, the average G‐force is 2.3 G when powered by the auxiliary engine and 2.8 G when powered by the nuclear motor. Tintin experiences a maximum G‐force of 3.0 G at the time the nuclear engine is turned on, lower than the maximum G‐force of 3.9 G felt by the Apollo 11 crew (Saturn Flight Evaluation Working Group, 1967).

Hence, this initial acceleration has led to a severe G‐LOC with no immediate response on a re‐established cardiovascular system. After reaching the escape velocity, the Moon Rocket moves with a constant velocity, thus the engine delivers a driving force equal to the gravitational force on the Moon Rocket. The total duration of G‐LOC in the entire crew is ∼12 min; prone or not, it is surprising that this occurs at an acceleration that is nowhere near 7.0 G, and, furthermore the crew do not display any neurological or cognitive deficits (apart from the habitual) upon regaining consciousness, although they must all have been subjected to >10 min of cerebral hypoperfusion! This raises the question of whether the repeated head trauma experienced by Tintin and his friends in previous adventures might have made them more susceptible to G‐LOC at relatively low accelerations, while also potentially reducing their susceptibility to concomitant neurological damage (Cyr et al., 2004). Repeated subconcussive head trauma, such as that sustained by professional boxers during sparring, is indeed associated with orthostatic intolerance owing to impaired dynamic cerebral autoregulation (Bailey et al., 2013). However, one might speculate that a form of cerebral ischaemic preconditioning is at play, by which repeated head trauma somehow reduces the susceptibility to neuronal damage in response to systemic hypotension and/or prolonged cerebral hypoperfusion. Beyond *The Adventures of Tintin* and other elements of popular culture, there is, in fact, some evidence of such a mechanism, given that the repeated cerebral ischaemia triggered by choking maneouvres seems to improve dynamic cerebral autoregulation in Brazilian Jiu‐Jitsu athletes (Stacey et al., 2021).

As the crew members continue their incredible adventure, an artificial gravitational field is seemingly generated by the Moon Rocket's rotation around its long axis, allowing them to walk around freely despite travelling at constant velocity outside Earth's gravitational field. In principle, this rotation should cause the crew to walk on the inside of the Moon Rocket's walls, as the centrifugal force pushes them outwards. However, the exact technology enabling this rotation remains unknown. As Thompson sits to observe the moon, he accidently pulls a switch, to which professor Calculus says, ‘Look what you've done you idiot! You've stopped the nuclear motor. The constant acceleration of our rocket created a sort of artificial gravity here inside…’. As this happens, weightlessness sets in, causing notable flushing in the crew members, reflecting the immediate shift of blood and fluids from the lower to the upper body, with concomitantly increased cardiac output and systemic vasodilatation documented in later studies (Norsk et al., 2006; Petersen et al., 2011), in addition to an increase in blood flow in the posterior cerebral and extracerebral circulation (Bailey et al., 2020; Lanéelle et al., 2023).

But why focus on comic book physiology at all? As an example, well‐researched works, such as *The Adventures of Tintin*, offer a fascinating window into the perception of space travel and related physiology at the time they were created. Tintin is rich in examples that prompt scientific discussion, such as whether drinking seawater can prevent dehydration when lost at sea, as depicted in *The Red Sea Sharks* (1958), or whether alcohol consumption is beneficial during hypothermia in *Tintin in Tibet* (1960). Such case studies from popular culture can, furthermore, be valuable tools in physiology education and science communication. As demonstrated convincingly by the Canadian neurophysiologist Paul Zehr (Zehr, 2014), these examples, even when somewhat scientifically inaccurate, might serve as relatable and recognizable touchpoints, providing a framework for understanding complex scientific concepts. Some of the authors of this piece have first‐hand experience of the power of this approach, when their systematic studies of Darth Vader's breathing in the *Star Wars* franchise (Berg & Plovsing, 2016; Plovsing & Berg, 2014) (paradoxically, a series notoriously inaccurate in its portrayal of both space travel and space physiology) were featured in *National Geographic* (Greshko, 2015), with subsequent unexpected attention from international media!

Hergé’s ‘Moon voyage duology’ began serialization in *Le Journal Tintin* in 1950. Remarkably, Tintin thus made his fictional Moon landing this year, 19 years before Neil Armstrong set foot on the lunar surface. Hergé himself humorously acknowledged this prophetic achievement (Figure 1). In fact, Tintin and his fellow crew members ventured into space a decade before human spaceflight officially began in 1961 with Russian cosmonaut Yuri Gagarin's orbital flight, followed shortly after by American astronaut Alan Shepard, igniting the famous space race between the Cold War superpowers. Even Snowy reached the Moon 7 years before the Soviet dog Laika was the first animal to orbit Earth aboard Sputnik 2 in 1957. Reading *Explorers on the Moon* in 1950, one could scarcely have imagined the real‐life parallels that would emerge. When Tintin exclaims, after taking his first steps on the lunar surface, ‘I have taken a few steps. For the first time certainly in the history of mankind, there is an explorer on the Moon!’, it is strikingly reminiscent of Neil Armstrong's immortal words nearly two decades later.

**FIGURE 1:**
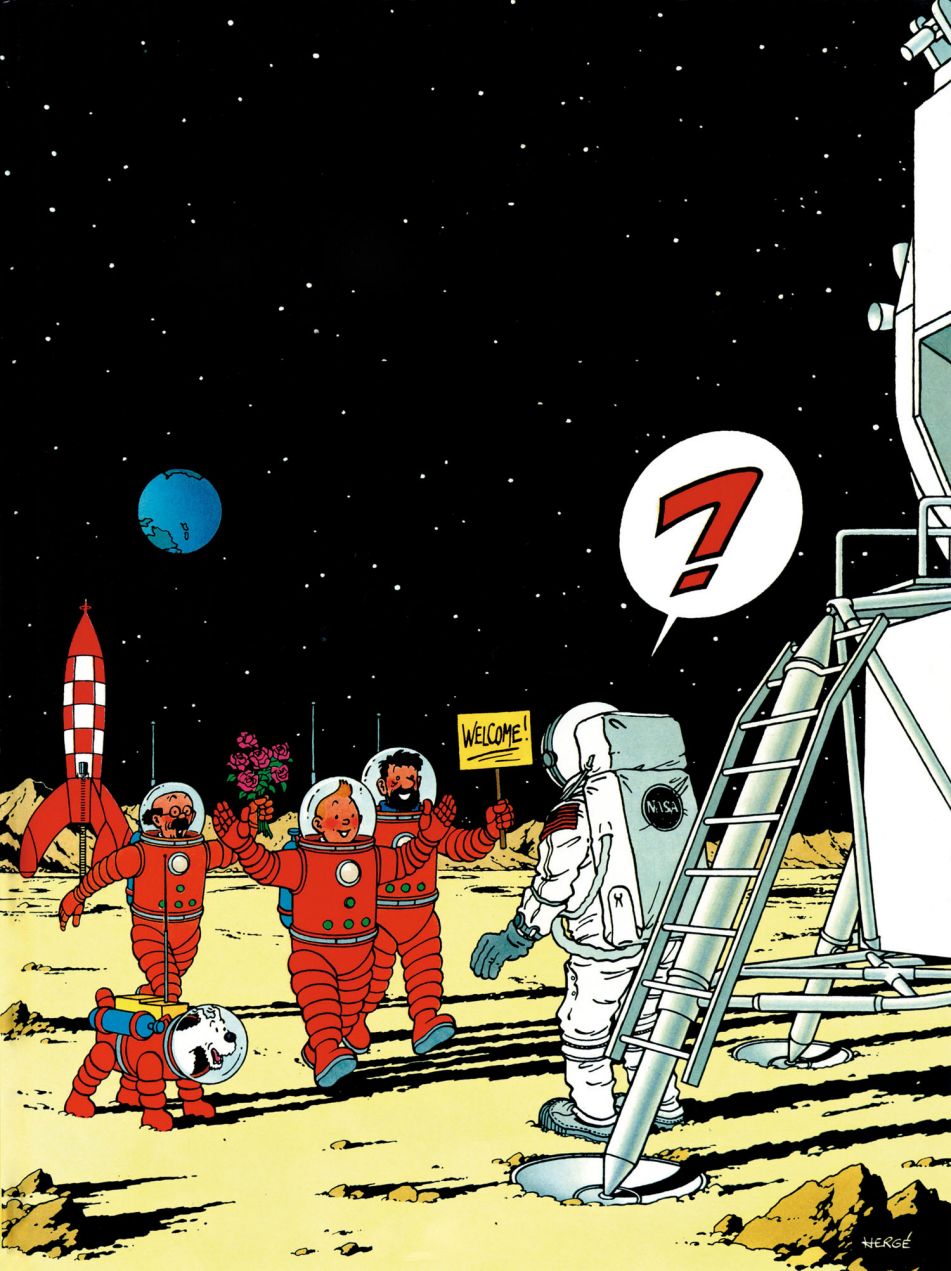
Tintin and friends welcome Neil Armstrong to the Moon. To celebrate the successful Moon landing by the Apollo 11 mission in the summer of 1969, Hergé sent Neil Armstrong this drawing. Reprinted with permission from Hergé/Tintinimaginatio 2025.

## AUTHOR CONTRIBUTIONS

Jacob P. Hartmann: First draft, data collection, data analysis, revisions. Mathis B. Mottelson: First draft, data collection, revisions. Rasmus H. Dahl: Data collection, revisions. Ronni R. Plovsing: Revisions. Ronan M. G. Berg: Conception, first draft, supervision, revisions. All authors approved the final version of the manuscript and agree to be accountable for all aspects of the work in ensuring that questions related to the accuracy or integrity of any part of the work are appropriately investigated and resolved. All persons designated as authors qualify for authorship, and all those who qualify for authorship are listed.

## CONFLICT OF INTEREST

None of the authors have any conflict of interest.

## FUNDING INFORMATION

The Centre for Physical Activity Research (CFAS) is supported by TrygFonden (grants ID 101390, ID 20045, ID 125132 and ID 177225). The funders had no role in study design, data collection and analysis, decision to publish or preparation of the manuscript.
